# Effect of Polyhedral Oligomeric Silsesquioxane on the Melting, Structure, and Mechanical Behavior of Polyoxymethylene

**DOI:** 10.3390/polym10020203

**Published:** 2018-02-17

**Authors:** Dorota Czarnecka-Komorowska, Tomasz Sterzynski

**Affiliations:** Poznan University of Technology, Institute of Materials Technology, Polymer Processing Division, Piotrowo 3 Street, PL-61138 Poznan, Poland; Tomasz.Sterzynski@put.poznan.pl

**Keywords:** polyoxymethylene (POM), octakis[(3-glycidoxypropyl)dimethylsiloxy]octasilsesquioxane (GPOSS), composites, morphology, mechanical properties

## Abstract

The effects of octakis[(3-glycidoxypropyl)dimethylsiloxy]octasilsesquioxane (GPOSS) on the crystallinity, crystal structure, morphology, and mechanical properties of polyoxymethylene (POM) and POM/GPOSS composites were investigated. The POM/GPOSS composites with varying concentrations of GPOSS nanoparticles (0.05–0.25 wt %) were prepared via melt blending. The structure of POM/GPOSS composites was characterized by differential scanning calorimetry (DSC), wide angle X-ray diffraction (WAXD), and polarized light microscopy (PLM). The mechanical properties were determined by standardized tensile tests. The morphology and dispersion of GPOSS nanoparticles in the POM matrix were investigated with scanning electron microscopy (SEM) and energy dispersive X-ray (EDX) analysis. It was observed that the dispersion of the GPOSS nanoparticles was uniform. Based on DSC studies, it was found that the melting temperature, lamellar thickness, and the degree of crystallinity of the POM/GPOSS composites increased. The POM/GPOSS composites showed an increased Young’s modulus and tensile strength. Finally, compared with the pure POM, the addition of GPOSS reduced the spherulites’ size and improved the crystallinity of the POM, which demonstrates that the nucleation effect of GPOSS is favorable for the mechanical properties of POM.

## 1. Introduction

Composites based on polyoxymethylene (POM), or polyacetal with silsesesquioxanes, are an interesting group of engineering materials due to the significant modification capabilities of these polymers and their wide application in industry. POM, with (–CH_2_–O–) as the main chain, is a major engineering plastic, with a high degree of mechanical strength, dimensional stability, and abrasion resistance. Two types of acetal are commercially available: homopolymers and copolymers [1].

Engineering applications require the creation of materials with significantly high strength properties and high thermal resistance. Thus, there is a need for research directed towards the modification of the structure and properties of materials, and such modification is the main aim of implementing various nanofillers. The nanoparticles incorporated into the polymer matrix usually significantly influence the polymer’s crystallization as well as the crystal phase type [2]. Particle size, their quantity, the degree of dispersion in the polymer matrix, and the processing conditions are the most relevant parameters in terms of composite properties [3,4].

Polyhedral oligomeric silsesquioxanes (POSS) are nanostructures with the empirical formula RSiO_1.5_, where R is a hydrogen atom or an organic group such as alkyl, alkylene, acrylate, hydroxyl, or epoxide units [4,5,6,7,8]. POSS may be referred to as a silica nanoparticle, consisting of a silica cage core and other organic functional groups attached to the corners of the cage [8]. The advantage of POSS as a nanofiller is due to the magnitude of its core; the core of octasilsesquioxanes can reach as high as approximately 0.5 nm. The whole molecule, depending on the substituent type, can range from 1 to 3 nm [9,10]. Comparing the average size of the macromolecular chains of these silsesquioxane molecule sizes, together with the possibility of implementation of the appropriate functional group, facilitates the deposition of POSS in the polymer matrix. Poly(methyl methacrylate) [10], polyethylene [11], polypropylene [12,13], poly(ethylene terephthalate) [14], polylactide [15], epoxide resins [16], polyurethanes [17], and polyoxymethylene [18,19] were all found to have an impact on certain physical properties such as glass transition, decomposition temperature, viscosity, fracture toughness, and barrier properties [20,21,22,23,24]. 

Few papers have been published concerning the effects of POSS molecules on the melting and crystallization, the microstructure, and the thermo-mechanical properties of polyoxymethylene [25,26,27]. Durmus et al. investigated the microstructure and isothermal melt-crystallization behavior of polyoxymethylene (POM) modified by methyl-polyhedral oligomeric silsesquioxanes (methyl-POSS) [25]. They pointed out that the addition of a PP/POSS nanocomposite dramatically enhances the crystallization rate of POM. It was concluded that the rate acceleration effect of the PP/POSS nanocomposite in POM crystallization is probably due to the tremendous increase in the number of nuclei due to the nano-POSS particles [25]. 

Lllescas et al. studied hybrid nanocomposites containing polyoxymethylene copolymer (POM) and four types of polyhedral oligomeric silsesquioxane (POSS) nanoparticles. They found that the added POSS nanoparticles can improve the thermo-mechanical properties of some polymers [26]. They reported that the formation of hydrogen bonding interactions between the POM and Si–OH groups of msib-POSS increased their mutual compatibility and led to nanometer-size dispersion of some msib-POSS molecules [26]. Pielichowski et al. studied the influence of the heating rate on the shape of the melting peak of polyoxymethylene (POM) using both high-speed differential scanning calorimetry (DSC) and StepScan DSC. They pointed out that a low heating rate facilitates the recrystallization of polyoxymethylene due to molecular nucleation [27].

The aim of this study was to determine the influence of a small amount of GPOSS on the crystallinity, crystal structure, morphology, and mechanical properties of POM, where the introduction of the nanoparticles is achieved via melt processing. 

## 2. Experimental Section

### 2.1. Materials

The commercial form of POM (Tarnoform 300), with a melt flow index of 0.9 g/10 min (190 °C, 2.16 kg), was supplied by Grupa Azoty S.A (Zakłady Azotowe, Tarnów-Moscice, Poland) for use in this study. GPOSS was synthesized by the hydrosilylation process [28], using a methodology developed at the Faculty of Organic Chemistry of Adam Mickiewicz University (Poznan, Poland). The synthesis of GPOSS was conducted in accordance with the procedure described in [29,30]. 

The chemical structure of GPOSS was confirmed by spectroscopic methods (NMR, FT-IR), and the results of analysis are as follows:

**^1^H NMR** (CDCl_3_, 298 K, 300 MHz) δ (ppm) = 0.05 (OSiCH_3_); 0.51 (SiCH_2_); 1.51(CH_2_); 2.47, 2.65 (CH_2_O); 3.00 (CHO); 3.25 (CH_2_O); 3.33, 3.56(OCH_2_).

**^13^C NMR** (CDCl_3_, 298 K, 75.5 MHz) δ (ppm) = −0.66 (SiCH_3_); 13.39 (SiCH_2_); 22.89 (CH_2_); 43.98 (CH_2_O); 50.29 (CHO); 71.75 (OCH_2_); 73.61 (CH_2_O).

**^29^Si NMR** (CDCl_3_, 298 K, 59.6 MHz) δ (ppm) = 12.87 (OSi(CH_3_)_2_); −109.13 (SiOSi).

**FT-IR** (ATR): 2998, 2955, 2931, 2869, 1253, 1070, 902, 838, 547 cm^−1^.

**Figure 1:**
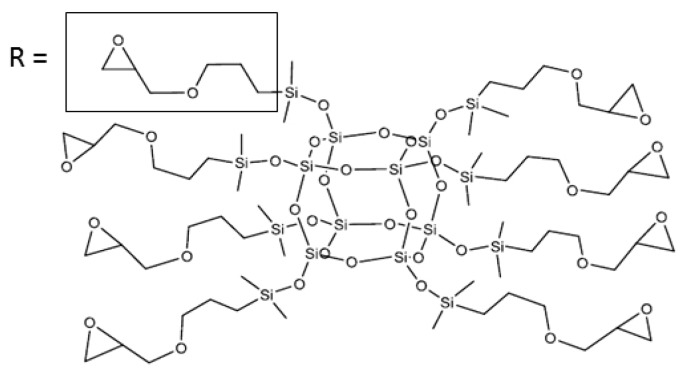
Chemical structure of the octakis[(3-glycidoxypropyl)dimethylsiloxy]octasilsesquioxane (GPOSS).

The chemical formula of GPOSS is presented in Figure 1.

### 2.2. Preparation of the POM/GPOSS Nanocomposites

POM pellets were ground into a powder using a Tria grinder and dried at 80 °C for 4 h. The POM powder was premixed with various amounts of GPOSS (0.05, 0.1, and 0.25 wt %) using the rotary mixer Retsch GM 200 (Retsch GmbH, Haan, Germany) (*t* = 4 min, *n* = 2000 rpm). The melt-mixing of the blends was carried out in a Brabender single screw extruder, equipped with an intense mixing zone, operating at 190 °C with a screw rotational speed of 15 rpm. The extruded rod was cooled in a water bath and subsequently pelletized. Standard test samples were produced by injection molding using an Engel machine (ES 80/20HLS, Schwertberg, Austria) with a 22 mm screw and an L/D ratio of 18. The processing parameters were as follows: injection temperature along the barrel: 180/190/200 °C; nozzle temperature: 210 °C; mold temperature: 60 °C.

### 2.3. Characterization

#### 2.3.1. Differential Scanning Calorimetry (DSC)

The melting and crystallization behavior of the composites were studied using a differential scanning calorimeter (Netzsch, DSC 204 F1 Phoenix, Selb, Germany) operating under nitrogen flow (150 mL/min). Samples of about 8 mg were heated up to 220 °C and held there in order to eliminate the thermal and mechanical prehistory. Next, the samples were cooled to 120 °C at a cooling rate of 20 °C/min and held there for 5 min and finally reheated again to 220 °C at the same rate. 

The degree of crystallinity (*X*_c_) of the samples was determined from the values of enthalpy melting registered during the second heating, using the following equation:

(1) $$X_{c}(\%)=\frac{\Delta H_{m}}{\left(1-\varphi \right)\Delta H_{m}^{0}}$$

where ∆*H*_m_ is the melting enthalpy of the samples, φ is the weight fraction of GPOSS nanofiller, and Δ$H_{m}^{0}$ is the melting enthalpy for a 100% crystalline of polymer—this latter measurement was taken as 186 J/g for the POM copolymer [31].

#### 2.3.2. Optical Polarized Light Microscopy (PLM)

An optical polarized light microscope (PLM) (Nikon Eclipse E400, Kanagawa, Japan), equipped with a Linkam THMS 600 hot stage (Linkam Scientific Instruments Ltd., Tadworth, UK) was used to study the crystallization process via cooling. The samples were heated at 200 °C for 2 min to obtain a full melting of the POM, then quickly cooled (20 °C/min) to the isothermal crystallization temperature of approximately 148 °C. The growth of the spherulites was observed during crystallization using an Opta-Tech camera (Opta-Tech, Warsaw, Poland) at 200× magnification.

#### 2.3.3. Wide-Angle X-ray Diffraction (WAXD)

The crystal structures of the POM and POM/GPOSS composites were evaluated with wide-angle X-ray diffraction (WAXD) measurements taken at room temperature, using a Brücker D2 diffractometer (Madison, Japan), equipped with Cu *K*α radiation (λ = 0.1542 nm), operating at 40 kV and 40 mA. Diffraction patterns were recorded for 2θ values ranging from 10 to 50°, at a scanning rate of 0.01°/s. The *d*-spacing of the POM/GPOSS was evaluated with the Bragg equation:

(2) 2dsinθ=nλ

where θ is the Bragg’s angle for the corresponding crystallographic plane (hkl), *n* is the order of diffraction, and λ is the incident wave length. 

#### 2.3.4. Mechanical Properties

The mechanical properties of the samples were determined by tensile tests performed using a universal testing machine: a Zwick/Roell Z005 (Ulm, Germany). The samples were dumbbell-shaped, with dimensions of 75 mm × 5 mm × 2 mm (test specimens type 1BA), according to the PN-EN ISO 527-2 standard. The tensile tests were run at room temperature. Tensile modulus was determined at a 1 mm/min cross head speed, whereas other tensile characteristics were measured at 50 mm/min speed. Young’s modulus (*E*), ultimate tensile strength (σ_M_), and tensile stress at break (σ_B_) were evaluated from the tensile stress-strain curves. The reported data were the average of the results of 10 specimens. 

#### 2.3.5. Scanning Electron Microscope (SEM)

The morphology of the fractured surfaces of the POM/GPOSS composite, coated with gold, was observed using a scanning electron microscope (SEM)—TESCAN TS 5135 (Brno, Czech Republic). The dispersion of GPOSS nanoparticles in the POM matrix was investigated using the back-scattered electron (BSE) signal and secondary electron (SE) signal, with an accelerating voltage of 15 kV, and the Si distribution was subsequently mapped.

## 3. Results and Discussion

### 3.1. Effect of GPOSS on Melting and Crystallinity

**Figure 2:**
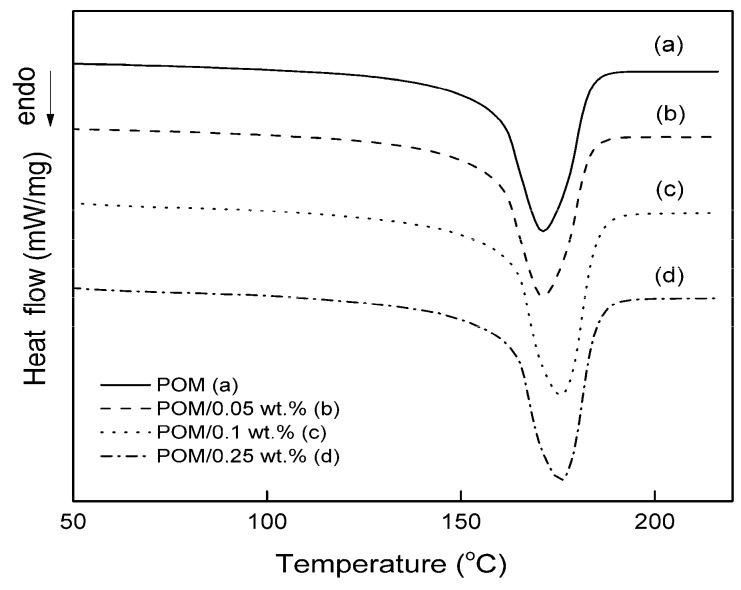
Differential scanning calorimetry (DSC) melting curves of pure POM (**a**) and its composites with different GPOSS concentrations at the first heating rate of 20 °C/min: (**b**) 0.05 wt %; (**c**) 0.1 wt %; (**d**) 0.25 wt %.

The DSC melting curves for pure POM and POM with GPOSS nanoparticles, registered during the first heating at a rate of 20 °C/min, are shown in Figure 2.

The onset of the melting temperature (*T*_m1 onset_), the maximum melting temperature (*T*_m1_), the melting enthalpy (∆*H*_m1_), the lamellar thickness (*l*_c_), the melting enthalpy (∆*H*_m2_), and the crystallinity (*X*_c_) are listed in Table 1. 

A clear single endothermal peak within the temperature range of 169–180 °C for the pure POM and its composites was observed, indicating that, as the result of modification, compatible blends of POM with GPOSS were achieved. This suggests a strong interfacial interaction between POM and the glycid groups of POSS. As seen in Figure 2, it follows that the melting temperature of POM/GPOSS composites becomes higher as GPOSS content increases. For example, an increase in the *T*_m_ from 169 °C in pure POM to 176 °C (an increase of approximately 7 °C) in POM with 0.25 wt % GPOSS was observed, an effect consistent with our primary observation for polyoxymethylene modified by vinyl groups of POSS [19].

The melting temperature (*T*_m1_) observed during the first DSC heating run may be related to the lamellar thickness, where a greater lamellar thickness corresponds to a higher polymer melting temperature. This relationship is described by the Gibbs-Thompson equation (Equation (3) [32]. Based on this equation, the lamellar thickness (*l*_c_) of the POM/GPOSS composites was evaluated and compared with the corresponding values of the pure POM.

(3) $$l_{c}=\frac{2\sigma_{e}\cdot T_{m}^{0}}{\Delta h_{0}(T_{m}^{0}-T_{m})}$$

where *T*_m_ is the observed melting temperature for a crystalline lamellar of thickness (*l*_c_), $T_{m}^{0}$ is the equilibrium melting temperature of the crystalline lamella of an infinite thickness, σ_e_ is the lamellar basal surface free energy (12.5 × 10^6^ J·cm^−2^ for POM) [33], and ∆*h*_0_ is the enthalpy of fusion for the crystalline phase (315 × 10^6^ J·cm^−3^ for POM) [34].

**Figure 3:**
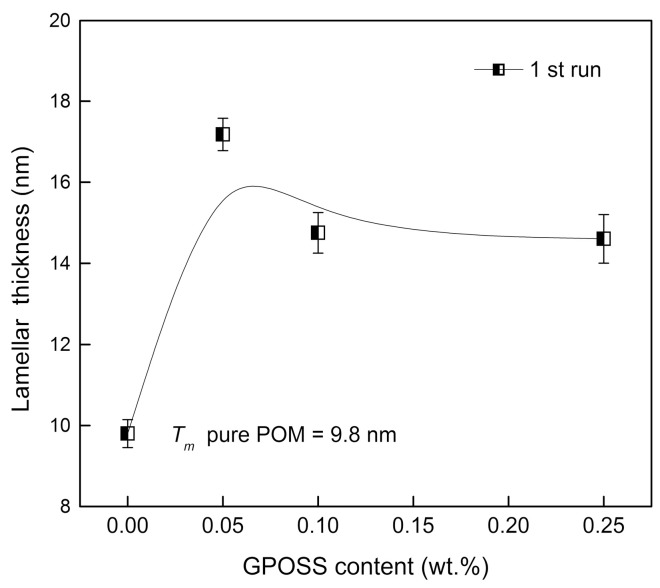
The variation in the lamellar thickness of pure POM and its composites, calculated from DSC (first run) experiments as a function of GPOSS content.

Figure 3 illustrates the relationship between the amount of GPOSS and lamellar thickness (*l*_c_) of the POM polymer single crystals. 

An increase in the lamellar thickness of POM with 0.25 wt % GPOSS to about 17 nm, compared with the pure POM where the lamellar thickness was equal to 9.8 nm, was noted. It is known that lamellar thickness increases with an increase in crystallization temperature [35]. A similar phenomenon has been observed in POM with carbon nanotubes (CNTs) [36].

As presented in Table 1, an increase in the *X*_c_ value from 72% in pure POM to 81% (i.e., an increase of about 13%) in POM modified with 0.25 wt % GPOSS was observed. A significant increase in enthalpy in the second melting (∆*H*_m2_) from 134.3 J/g in pure POM to 150 J/g in the highest amount of POM/0.25 wt % GPOSS may indicate that GPOSS additives play a heterogenic nucleation role in POM. 

Similar effects have been observed by Hu and Ye through the analysis of the POM crystallization process with the addition of polyamide 6 (PA6). It has been shown that PA6 leads to an increase in the degree of crystallinity (*X*_c_), improving the crystallization growth rate and resulting in a reduction in the size of spherulites in POM [37].

### 3.2. Effect of GPOSS on the Spherulitic Morphology

**Figure 4:**
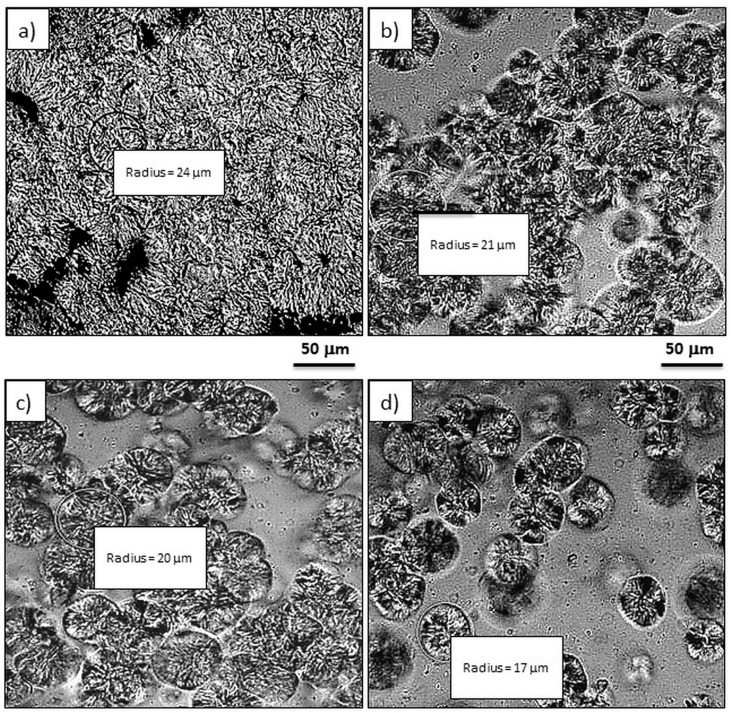
PLM micrographs of the crystallized pure POM (**a**) and its composites with different GPOSS concentrations: (**b**) 0.05 wt %; (**c**) 0.1 wt %; (**d**) 0.25 wt %. *T* = 148 °C. The cooling rate was set to 20 °C/min. (magnification 200×).

The spherulitic morphology of pure POM and POM/GPOSS composites was observed using optical polarized light microscopy (PLM), as shown in Figure 4. 

**Figure 5:**
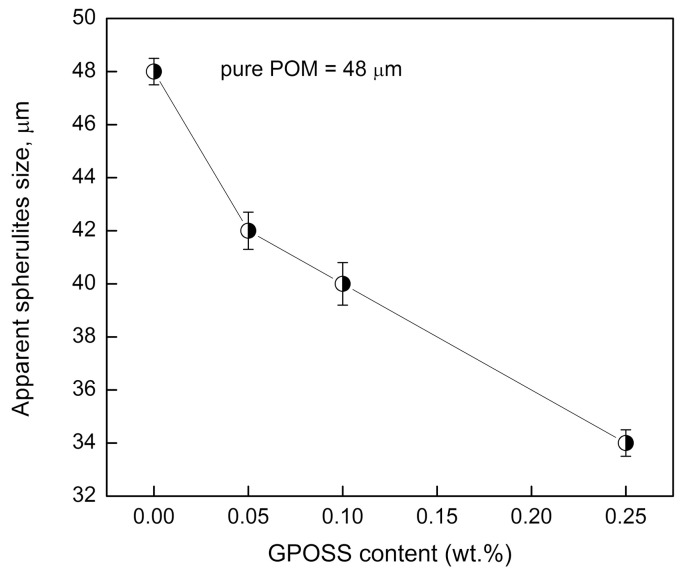
The dependence of the size of spherulites in pure POM and its composites with different GPOSS concentrations. The cooling rate was set to 20 °C/min.

Significant differences between the structure of pure POM (Figure 4a) and POM modified with GPOSS can be seen in Figure 4b–d. For all cases, spherulite forms differing in magnitude, shape, spatial distribution, and well-developed spherulitic morphology can clearly be seen. It is obvious that the POM spherulites become smaller as GPOSS content increases (Figure 4b–d). Figure 5 illustrates the dependence of the size of spherulites in the POM on GPOSS content; the addition of 0.25 wt % GPOSS leads to a significant decrease in POM spherulite size—specifically, from 48 × 10^−6^ to about 35 × 10^−6^ m. Such an effect is due to the heterogeneous nucleation role of GPOSS.

### 3.3. Effect of GPOSS on the Crystal Structure

**Figure 6:**
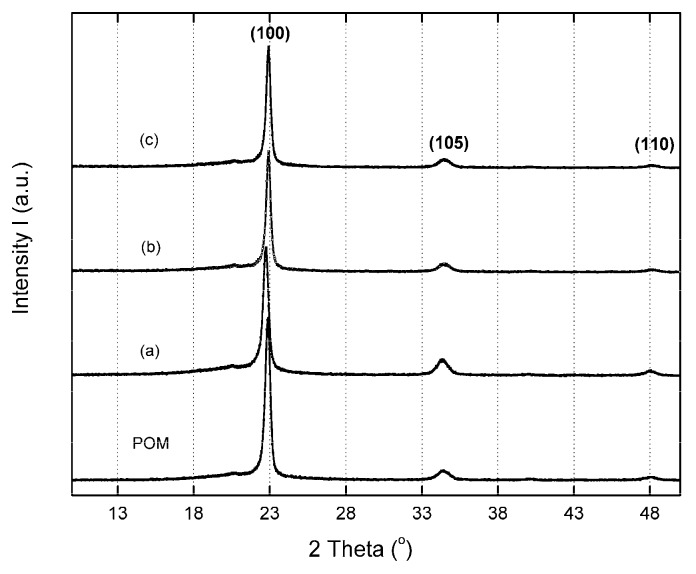
WAXD curves of pure POM and its composites with different GPOSS concentrations at room temperature: (**a**) 0.05 wt %; (**b**) 0.1 wt %; (**c**) 0.25 wt %.

The crystal structure of POM was studied using the WAXD technique. The analysis of the WAXD allows us to determine the crystal atom structure, including the position/symmetry of the atoms in the unit cell, the unit cell size, and the shape/size of the nanocrystalline domain [38]. The X-ray diffraction patterns for the POM and POM/GPOSS composites with different GPOSS concentrations, as a function of Bragg’s angle (2θ) and measured at room temperature, are presented in Figure 6. 

As can be seen in Figure 6, two intense Bragg diffraction peaks can clearly be observed at the Bragg angles 2θ = 22.7° and 2θ = 34.14°, where the peak at 22.7° corresponds to the basal reflection (100), and the peak at 34.14° corresponds to the basal reflection (105). POM crystallizes in a hexagonal crystallographic form with unit cell dimensions of a = b = 4.46 Å and c = 17.3 Å [39]. In general, it was observed that the X-ray diffraction patterns of POM/GPOSS composites are similar to the diffraction pattern of the hexagonal form observed for the crystalline phase of pure POM. The crystallite size (*L*_hkl_), according to the Scherrer formula (Equation (3)), was evaluated for the crystallographic plane (100), using the following equation:

(4) $$L_{\mathrm{hkl}}=\frac{K\lambda }{\beta_{\mathrm{hkl}}\times \cos\theta_{\mathrm{hkl}}}$$

where *L*_hkl_ is the apparent crystallite size of the normal direction of the {hkl} crystal plane (in Angstrom, 1 Å = 10^−10^ m), *K* is a Scherrer constant, normally taken as 0.9, β_hkl_ is the full-width at half-maximum (in radian) of the crystalline peak, nm, θ is the Bragg diffraction angle, and λ is the wavelength of entrance X-ray (0.154 nm for Cu), as presented in Table 2.

The highest WAXD diffractogram intensity was observed for the POM/GPOSS composites, indicating the higher crystalline structure order of the POM. 

Based on WAXD studies, it was also shown that, with an increase in GPOSS content in the POM, an increase in the diffraction peak at half height width occurs, corresponding to a reduction in crystal size (from 228 to 143 Å). 

In 0.05 and 0.25 wt % POSS, a clear decrease in the L_100_ crystallite size of composites, in comparison with pure POM, was found, suggesting that GPOSS has a nucleating effect on POM. 

### 3.4. Effect of GPOSS on Mechanical Behaviour

**Figure 7:**
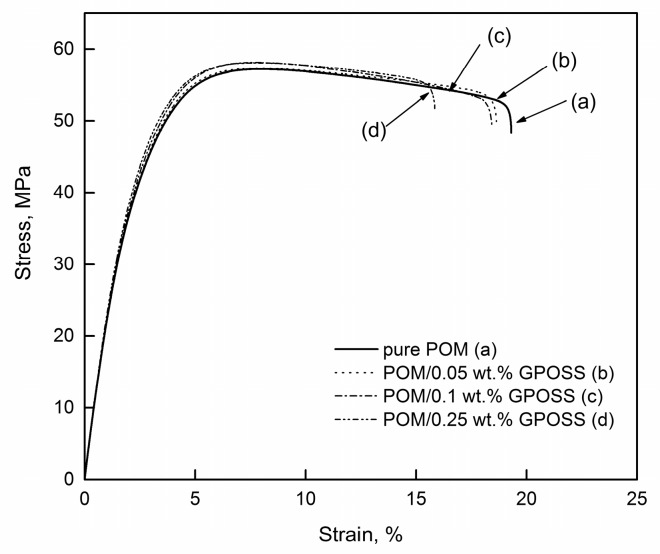
Tensile stress-strain curves of pure POM (**a**) and its composite with different GPOSS concentrations: (**b**) 0.05 wt %; (**c**) 0.1 wt %; (**d**) 0.25 wt %.

In Figure 7, typical tensile stress-strain curves of pure POM and POM/GPOSS composites with different GPOSS nanoparticle concentrations are presented. 

The average values and standard deviations of mechanical properties are given in Table 3.

The Young’s modulus (*E*), ultimate tensile strength (σ_M_), and tensile stress at break (σ_B_) values of POM/GPOSS composites and pure POM were compared, and an increase in tensile properties, such as strength and modulus, with increasing GPOSS concentrations was noted. The Young’s modulus of the POM was increased from 1.8 to 2.6 GPa with the addition of 0.25 wt % GPOSS, which corresponds to an approximate 45% increase. Similarly, an increase in tensile strength was observed for POM/GPOSS composites compared to pure POM. The maximum value of the tensile strength (σ_M_) was observed for the POM/GPOSS composite with the addition of 0.25 wt % GPOSS. Compared with the pure POM, the addition of GPOSS reduced the size of the spherulites and improved the crystallinity (*X*_c_) of the POM, which demonstrates the nucleation effect of GPOSS, as the nucleus is favorable for the mechanical properties. These results are in agreement with the DSC results. The changes in mechanical properties may also be a result of a good dispersion of GPOSS nanoparticles in the POM matrix and a good adhesion of these two components. 

### 3.5. Effect of GPOSS on Morphology

**Figure 8:**
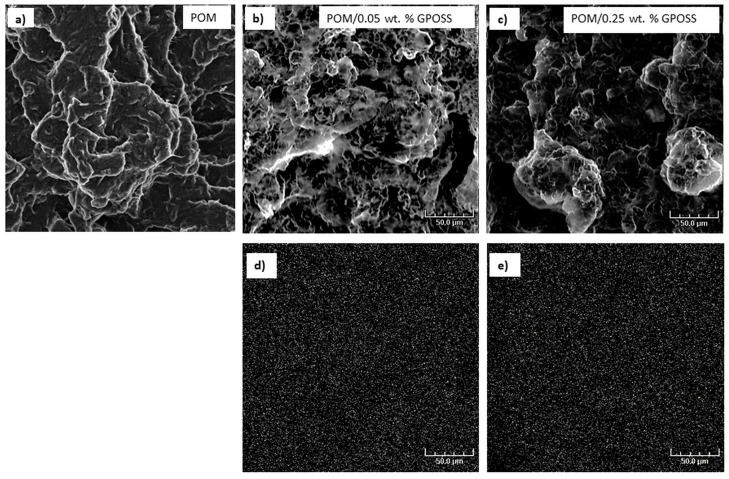
SEM images of the fractured surface (**a**–**c**) and Si mapping (**d**–**e**) of pure POM (**a**) and its composites with different GPOSS concentrations: (**b**) 0.05 wt %; (**c**) 0.25 wt %.

SEM microscopy was used to analyze the morphology and dispersion of particles of GPOSS in the POM matrix. Figure 8 presents SEM images that show the morphologies of pure POM (a) and POM/GPOSS composites with the addition of 0.05 wt % (b) and 0.25 wt % GPOSS (c).

A relatively homogenous distribution of GPOSS particles in the POM matrix can be seen in Figure 8a–c. The dispersion of the GPOSS particles in the POM matrix was homogeneous, and a similar phenomenon was described in the literature for silsequioxanes with polyamide and polyethylene [30]. Figure 8d,e shows the energy dispersive X-ray spectrometer (EDS) composition distribution maps of the Si element on the surface of the POM/0.05 wt % GPOSS (d) and POM/0.25 wt % GPOSS composites (e). The white dots in this figure are the X radial signals radiating from the Si element. A random dispersion of 0.25 wt % GPOSS nanoparticles was found on the entire fractured surface of the POM. A similar effect was observed for polyoxymethylene and POM/HAp nanocomposites [40]. 

## 4. Conclusions

In the study reported herein, the effect of GPOSS on the melting behavior, structure, and mechanical properties of POM was investigated. The properties of POM/GPOSS composites were characterized as a function of the GPOSS content, which varied from 0 to 0.25 wt %. DSC measurements indicated a significant change in the melting temperature and crystallinity of the nanocomposites, relative to the matrix. POM/GPOSS composites of an evidently higher degree of crystallinity were achieved, indicating a heterogenic nucleation role of the GPOSS additive for pure POM. 

Polarized microscopy results indicate that the POM spherulites become smaller as GPOSS content increases. During POM crystallization, small spherulites about 35 μm in diameter were produced. The incorporation of GPOSS reduced the mean size of the spherulites, suggesting a nucleation effect. X-ray diffraction results indicate no significant changes in the hexagonal structure of the POM in the presence of GPOSS. 

The enhancement in the Young’s modulus of the composites containing 0.25 wt % GPOSS, relative to the pure POM, was about 45%. This suggests that GPOSS should work as a reinforcement in the POM matrix. The reason for this is due to the good dispersion of GPOSS in the POM matrix. SEM results proved the existence of a uniform structure in the heterogeneous compatible blends, in which intensive interfacial interactions may be present.

## Figures and Tables

**Table 1:** DSC data of pure POM and POM/GPOSS composites (cooling and heating rate: 20°/min).

Samples	*T*_m1_ (°C)	*T*_m1 onset_ (°C)	∆*H*_m1_ (J/g)	*l*_c_ (nm)	∆*H*_m2_ (J/g)	*X*_c_ (%)
pure POM	169.2	158.0	−138.5	9.8	−134.3	72
POM/0.05 wt % GPOSS	171.2	159.0	−142.0	11.4	−135.8	73
POM/0.1 wt % GPOSS	173.7	160.0	−143.5	14.2	−137.4	74
POM/0.25 wt % GPOSS	175.7	163.2	−154.1	17.2	−149.9	81

**Table 2:** Crystal size (*L*_hkl_) evaluated according to the Scherrer formula.

Samples	L_100_ (Å)
pure POM	228
POM/0.05 wt % GPOSS	217
POM/0.1 wt % GPOSS	186
POM/0.25 wt % GPOSS	143

**Table 3:** Mechanical properties, with standard deviations, of pure POM and POM/GPOSS composites.

Samples	Ultimate tensile strength (MPa)	Young’s modulus (GPa)	Tensile stress at break (MPa)
pure POM	55.00 ± 0.52	1.83 ± 0.13	45.97 ± 0.65
POM/0.05 wt % GPOSS	57.50 ± 0.43	2.44 ± 0.23	49.78 ± 0.78
POM/0.1 wt % GPOSS	57.64 ± 0.90	2.48 ± 0.15	49.90 ± 0.69
POM/0.25 wt % GPOSS	57.80 ± 0.95	2.60 ± 0.25	50.25 ± 0.73
